# Potential role of tea drinking in preventing hyperuricaemia in rats: biochemical and molecular evidence

**DOI:** 10.1186/s13020-022-00664-x

**Published:** 2022-09-15

**Authors:** Siyao Sang, Lufei Wang, Taotao Liang, Mingjie Su, Hui Li

**Affiliations:** 1 State Key Laboratory of Genetic Engineering, School of Life Sciences, Fudan University, Shanghai, 200438 China; 2 MOE Key Laboratory of Contemporary Anthropology, Fudan University, 200438 Shanghai, China; 3 Department of Haematology, Affiliated Cancer Hospital of Zhengzhou University, Henan Tumour Hospital, Zhengzhou, 450008 China; 4 Human Phenome Institute, Fudan University, 200438 Shanghai, China; 5 Fudan-Datong Institute of Chinese Origin, Shanxi Academy of Advanced Research and Innovation, 037006 Datong, China

**Keywords:** Tea, Hyperuricaemia, Uric acid, Kidney injury, Inflammation

## Abstract

**Background:**

Lifestyle and diet play a significant role in hyperuricaemia. Accumulating evidence indicates that tea consumption is associated with hyperuricaemia and gout. However, diverse compounds in different types of tea make it quite difficult to determine the relevant molecular mechanism. Here, we compared the effects of six types of tea on hyperuricaemia induced by potassium oxonate (PO) and hypoxanthine in rats and investigated the possible underlying mechanisms.

**Methods:**

Rats were randomly assigned to ten groups: the control, hyperuricaemia model, benzbromarone positive control, traditional Chinese medicine *Simiao San* positive control, green tea, yellow tea, black tea, white tea, red tea, and cyan tea treatment groups. After 21 days, uric acid (UA), xanthine oxidase (XOD), alanine aminotransferase (ALT),blood urea nitrogen (BUN), and creatinine (CRE) were assessed. Serum levels of interleukin-1β (IL-1β) were measured with an enzyme-linked immunosorbent assay. Haematoxylin–eosin staining and immunohistochemistry were used to assess liver and kidney injury.

**Results:**

The levels of UA, CRE, and BUN in the treatment group were decreased to varying degrees. There was a significant reduction in UA, CRE, and BUN levels for yellow tea compared to the positive control drugs. Yellow tea suppressed XOD activity and alleviated hepatic and kidney injury. Network pharmacology and untargeted metabolomics indicated that ten yellow tea bioactive ingredients and 35 targets were responsible for preventing hyperuricaemia, which was mediated by 94 signalling pathways, including IL-1β and TNF.

**Conclusion:**

These findings indicate that green tea cannot reduce the serum uric acid level of hyperuricaemic rats. Yellow tea can significantly improve hyperuricaemia by regulating the inflammatory response, autophagy, and apoptosis. This study provides a potential candidate for the treatment of hyperuricaemia and a basis for selecting therapeutic tea for patients with hyperuricaemia.

**Supplementary Information:**

The online version contains supplementary material available at 10.1186/s13020-022-00664-x.

## Background

Hyperuricaemia and gout are chronic diseases of urate crystal deposition, accompanied by elevated serum uric acid concentrations and monosodium urate precipitation in joints and other tissues[1]. Disorders of purine metabolism cause hyperuricaemia and its prevalence is affected by multiple factors, such as heredity, sex, age, lifestyle, dietary habits, drug treatment, and economic development [2, 3]. Recent epidemiological studies have shown that hyperuricaemia may be involved in hypertension, diabetes, atherosclerosis, chronic kidney disease, atrial fibrillation (AF), and the occurrence of cardiovascular events [4–6]. The prevalence of hyperuricaemia was found to be 20.2% in men and 20.0% in women [7]. The prevalence of hyperuricaemia increases with age, reaching its highest at 27.8% in people aged 80 years and older. With changes in people's dietary structure, the prevalence of hyperuricaemia is increasing year by year. Over the past 5–10 years, much progress has been made in understanding the pathophysiology and genetics of hyperuricaemia and gout, but the quality of care remains a major challenge in gout management [8].

Uric acid is a metabolite of hypoxanthine and xanthine oxidized by xanthine oxidase (XOD) in the liver [9, 10]. It is filtered by the glomerulus, then reabsorbed by the renal tubules, and finally excreted from the body through the bladder. The excessive production of uric acid from purine metabolism in the liver or a reduction of the filtration effect of the kidney can lead to the deposition of urate crystals and hypoxanthine [4]. Hyperuricaemia can induce diabetes, hypertension, myocardial infarction, and other diseases.

There are many feasible drugs for treating gout. The first-line urate-lowering medications for chronic gout are the xanthine oxidase inhibitors–allopurinol and febuxostat [11]. Colchicine is used both during acute episodes and in chronic maintenance therapy [12]. Benzbromarone and probenecid treat hyperuricaemia and gout by inhibiting renal reabsorption of uric acid [13, 14]. The therapeutic effect of these drugs is not ideal, and they can cause organ damage, are not feasible for long-term use, and have high medical costs. Currently, there are traditional Chinese medicines (TCMs) such as Simiao San, that are used as supplements to treat hyperuricaemia [15]. Simiao San has been widely used for thousands of years for its safety and efficacy in the treatment of gouty arthritis and hyperuricaemia. The uric acid-lowering effects of traditional Chinese medicines are not strictly independent, and some herbal medicines have more than one effect. Experiments have shown that TCM is able to mitigate hyperuricaemia in various murine models, by inhibiting XOD activity, modulating urate transporters, and exerting anti-inflammation and antioxidative effect [15–17].The side effects of TCM in the treatment of hyperuricaemia are unclear. Given the potential impact of gout and hyperuricaemia on cardiovascular and metabolic diseases, there is an urgent need to find safe and effective intervention treatments.

Tea, brewed from the leaf of *Camellia sinensis*, is the second most consumed beverage in the world after water and may be associated with hyperuricaemia and gout risk. There are thousands of tea production processes around the world. In 1979, Chinese tea scientists analysed the production procedures and chemical components of various types of teas and successfully divided them into six categories: green tea, cyantea, redtea (commonly known as black tea), white tea, black tea (commonly known as dark tea), and yellow tea [18]. The finding of A. Dankowska and W. Kowalewski shows that black, green, white, yellow, red and cyan teas produced by different methods have different Ultra violet–visible spectroscopy, fluorescence and near-infrared spectroscopy spectral characteristics [19]. The chemical composition of tea is complex: polyphenols, amino acids, flavonoids, proteins, volatile compounds, fluorides, minerals and trace elements, and other indeterminate compounds [20, 21]. Over the past several decades, teas and their metabolites have been intensively investigated for their wide-ranging beneficial health effects in preventing and alleviating resistance to metabolic disorders, cancer, oxidation, and inflammation [18]. The functions of green tea have been widely reported [22–24]. Green tea inhibits brain aging by activating nerve cells through EGCG and its degradation products, and by reducing stress with theanine and arginine [25]. Black tea has beneficial effects on glucose homeostasis and improved insulin resistance in patients with type 2 diabetes [26]. Red and cyan teas have been reported to possess strong antioxidant activities [27, 28]. White tea has antibacterial and anti-inflammatory properties [29]. Yellow tea is a little-known tea with few reports on its beneficial effects [30–32].Research on the effects of tea on hyperuricaemia has focused on red and green tea [33, 34]. Some researchers have come to conflicting conclusions about the same tea. A recent study showed that daily drinkers of green tea exhibited a dose-dependent and statistically significant fourfold increased rate of hyperuricaemia [35]. Nevertheless, an earlier study showed that green tea drinking did not lead to a statistically significant change inserum uric acid [36]. Therefore, we explored the effect of tea drinking on hyperuricaemia, especially in the liver and kidney. Then, we used liquid chromatography tandem mass spectrometry (LC–MS/MS) and a network pharmacology system to identify and analyse the effective active ingredients and potential targets of tea and explored their possible mechanisms of action.

## Methods

### Chemicals and reagents

Hypoxanthine and monosodium urate (MSU) were purchased from Sigma–Aldrich (St. Louis, MO, USA). Potassium oxonate (PO) was purchased from Adamas Reagent Co.Ltd. (Shanghai, China).Assay kits for serum ALT, UA, BUN, CRE, and XOD were purchased from Nanjing Jian Cheng Bioengineering Institute (Nanjing, China). Enzyme-linked immune sorbent assay (ELISA) kits for IL-1β were purchased from DAKEWE (Beijing, China). All antibodies were obtained from Abcam (Cambridge, UK).

Turquoisepearls (green tea), golden bricks (yellow tea), golden fungi (black tea), red garment (cyan tea), old eyebrows (white tea), and red grape (red tea) were purchased from Bud-Chem Tea Co. Ltd. (Jiangkou, Guizhou, China).

### Tea preparation

Green tea, cyan tea, and red tea were brewed with 80 °C, 95 °C, and 99 °C water for 2–5 min, respectively. White tea, yellow tea, and black tea were boiled for 15–20 min to make the tea. All final tea preparations had a concentration of 0.3 g/mL. The detailed protocols for the tea preparation were as described previously[37].

### Animal study

Male Sprague–Dawley (SD) rats (body weight 200 ± 20 g) were purchased from Shanghai Slac Laboratory Animal Co. Ltd. (Certificate No.: 20170005059225). The rats were maintained in specific pathogen-free conditions, with a 12 h light/dark cycle at 20–22 °C and 45 ± 5%humidity. All rats were randomly assigned to ten groups: (a) control group (Con, n = 10), (b) hyperuricaemia and gout model group (Veh, n = 10), (c) benzbromarone treatment group (Ben, n = 10, 4 mg/kg/day), (d) TCM Simiao San treatment group (SMS, n = 10, 480 mg/kg/day), (e) green tea treatment group (G, n = 10,10 mL/kg/day), (f) yellow tea treatment group (Y, n = 10, 10 mL/kg/day), (g) black tea treatment group (B, n = 10, 10 mL/kg/day), (h) white tea treatment group (W, n = 10, 10 mL/kg/day), (i) red tea treatment group (R, n = 10, 10 mL/kg/day), and (j) cyan tea treatment group (C, n = 10, 10 mL/kg/day). Except for the Con group, rats in each group were intraperitoneally injected PO (300 mg/kg/day) with a: hypoxanthine (300 mg/kg/day) suspension once a day. The rats in the Con group were treated with PBS according to the same schedule. Groups (c)–(j) were gavaged with the prepared tea at a concentration of 0.3 g/mL once a day, and the Con and Veh groups were treated similarly with water. On Day 21, the rats were killed after anesthetization, and blood samples were collected by cardiac puncture. The kidney and liver were removed and fixed in 4% paraformaldehyde.

### Histological analyses

For histological evaluation, the right kidneys and livers were fixed with 4% paraformaldehyde and embedded in paraffin. The kidneys and livers were cut into 3-μm sections and stained with haematoxylin and eosin for general morphological analysis and Masson staining for collagen deposition and interstitial lesion assessment. The sections were photographed under a light microscope and analysed by a pathologist blinded to the animal groups and drug treatments.

### Immunohistochemistry (IHC)

The expression of NLRP3 and F4/80 in the kidney and liver was evaluated by IHC staining. For antigen retrieval, 4-μm-thick paraffin sections were placed in citrate buffer and microwaved with medium heat for 8 min and then allowed to stand for 8 min, followed by moderate-low heat for 7 min. After blocking with 3% bovine serum albumin, the sections were incubated with anti-NLRP3, and anti-F4/80 antibodies. Next, they were incubated with the appropriate secondary antibody. Finally, the sections were stained with diaminobenzidine and counterstained with haematoxylin. Positive staining was visible a brown–yellow under a microscope (Eclipse Ci-L, Nikon, Tokyo, Japan).

### Enzyme-linked immunosorbent assay (ELISA)

We assayed the serum contents of IL-1β with ELISA kits. The details of the ELISA have been described previously [38].

### ALT, XOD, BUN, CRE and UA activity assay

The blood samples were kept at 4 °C for 4 h to clot and then centrifuged at 2500×*g* for 10 min to obtain the serum. Then, the ALT, XOD, BUN, CRE and UA activities in the serum were determined using assay kits (Nanjing Jiancheng Bioengineering Institute, Nanjing, China) according to the manufacturer's guidelines.

### LC–MS-based untargeted metabolomics analysis

The prepared teas were analysed with an LC***–***MS/MS system, a Waters 2D ultra-performance liquid chromatograph (Waters, USA) coupled with a high-resolution mass spectrometer Q Exactive HF (Thermo Fisher Scientific, USA). Chromatographic separation was performed using the Hypersil GOLDaQ column (100 × 2.1 mm, 19 μm, Thermo Fisher Scientific, USA). The column temperature and detection wavelength were set at 40 °C, and the injection volume was set to 5 μL. The mobile phase consisted of 0.1% formic acid in 100% water (A) and 0.1% formic acid in 100% acetonitrile (B) at a flow rate of 5 μL/s. The linear gradient elution was as follows: 0–2 min, 5% B; 2–22 min, 5–95%; 22–27 min, hold at 95%; 27–30 min, decrease to 5%.

The raw data for MS1 and MS2 were acquired from a Q Exactive mass spectrometer (Thermo Fisher Scientific, USA). The parameters of the electrospray source under negative ionization were as follows: spray voltage, 3.2 kV (ESI−); capillary temperature, 320 °C; aux gas heater temperature, 350 °C; and m/zrange, 150–1500 m/z. The parameters of the MS1 scan were as follows: resolution (70,000), automatic gain control (AGC) target (1.0 × 10^6^), and maximum injection time (100 ms). For the data-dependent MS2, the following parameters were used: resolution (35,000), AGC target (2.0 × 10^5^), and maximum injection time (50 ms). The stepped normalized collision energy (NCE) values were set at 20, 40, and 60 eV. Each sample was tested in six repetitions.

The raw data were processed by Compound Discoverer 3.1 (Thermo Fisher Scientific, USA), including peak extraction, alignment, and quantification. Compound identification was verified by authentic standards and MS/MS fragmentations. Meanwhile, PubMed and HMDB were also used to identify the compounds.

### Gene set of the identified compounds in yellow tea and hyperuricaemia

The identified compounds were filtered using ADMET properties. ADME parameters were acquired from the TCMSP (https://tcmsp-e.com/), and used to evaluate the chemicals' absorption, distribution, metabolism, and excretion. The yellow tea compounds were filtered based on the criteria of oral bioavailability (OB) ≥ 30% and drug-likeness ≥ 0.18. After initial filtering, the gene set of the bioactive compounds was collected from the TCMSP, and HERB databases (http://herb.ac.cn/).

Genes related to the treatment and prevention of hyperuricaemia were collected from two databases: GeneCards (https://www.genecards.org/) and DisGeNet (https://www.disgenet.org/). Gene sets obtained from taking the intersection between yellow tea (YT) compound-related genes and antihyperuricaemia-related genes were used for further analysis.

### Construction of the PPI network and compound-target network

The STRING database was used to construct the protein–protein interaction network for the overlapping targets between hyperuricaemia and yellow tea. The species “*Homo sapiens*” was selected. Meanwhile, the interaction network of the yellow tea compounds and the overlapping targets was established using Cytoscape 3.9.0 software. Crucial targets were identified using molecular complex detection (MCODE) (a plugin in Cytoscape).

### Enrichment analysis of GO and KEGG pathways

Gene ontology (GO) and Kyoto Encyclopedia of Genes and Genomes (KEGG) pathway analyses were used to unveil the potential molecular mechanism and the critical signalling pathways. The “ClusterProfile” package was used to perform enrichment analysis for GO and KEGG pathways in R software version 3.4.0.

### Statistical analyses

The values are expressed as mean ± s.e.m. The unpaired t-test (GraphPad Prism 9 Software) was used for statistical analysis. Data points were not excluded. The researchers involved in this study were not blinded during sample collection or data analysis. The sample size was selected based on the preliminary results to ensure sufficient power. *P* values < 0.05 were considered significant.

## Results

### Effect of tea on the UA, CRE, and BUN levels of rats with hyperuricaemia

**Fig. 1:**
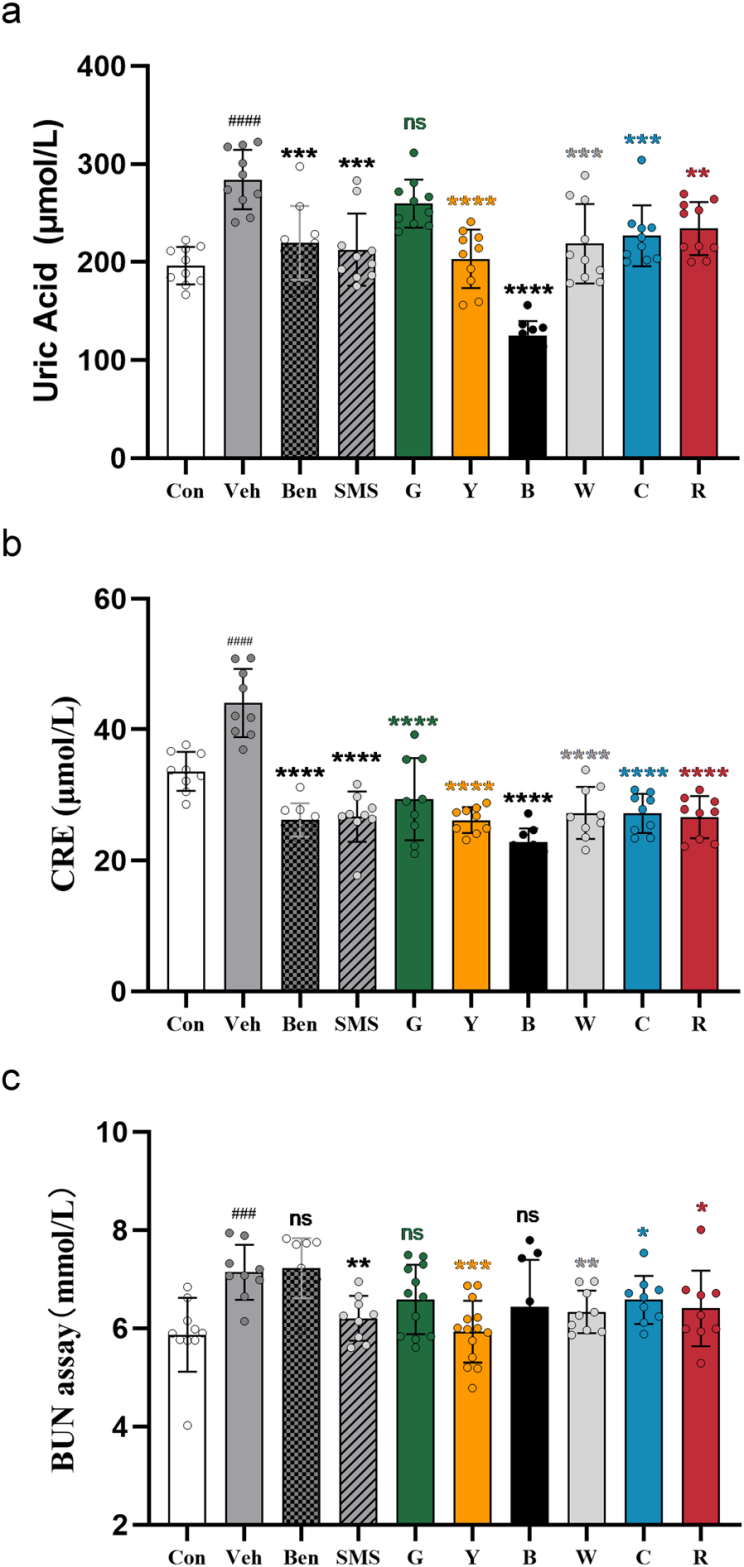
Effects of tea on UA, CRE, and BUN levels in PO- and hypoxanthine-induced hyperuricaemia in rats. Data were given as themean ± SD (n = 10). Ben, benzbromarone (4 mg/kg/day) treatment group; SMS, traditional Chinese Medicine Simiao San (480 mg/kg/day) treatment group; G, green tea (10 mL/kg/day) treatment group; Y, yellow tea (10 mL/kg/day) treatment group; B, black tea (10 mL/kg/day) treatment group; W, white tea (10 mL/kg/day) treatment group; C, cyan tea (10 mL/kg/day) treatment group; R, red tea (10 mL/kg/day) treatment group; ^#^Represents significant difference compared with the Con group, ^###^*P* < 0.001; *Represents significant difference compared with the Veh group, *P < 0.05, **P < 0.01, ***P < 0.001, ****P < 0.0001

Excessive production or insufficient excretion of UA can cause kidney damage. As shown in Fig. 1a, there was no difference in UA levels between the green tea treatment group and the Veh group. In contrast, the other types of tea treatment markedly reduced the serum UA levels in rats with hyperuricaemia. The UA levels of the yellow tea and black tea treatment groups were remarkably lower than those of the Vehgroup (*P* < 0.0001).

The level of CRE in the serum reflects the filtration function of the glomerulus. As shown in Fig. 1b, all types of tea decreased the level of CRE to restore the filtration function of the glomerulus, similar to the positive control groups (Ben group and SMS group).

BUN is also an important indicator of kidney injury. As shown in Fig. 1c, the BUN level of the Veh group was significantly higher than that of the Con group. Compared with the Veh group, TCM Simiao San reduced the BUN level in the serum of hyperuricaemic rats. The effect of white tea was similar to that of Simiao San (*P* < 0.001). Compared with the Vehgroup, red tea and cyan tea slightly reduced the BUN levels in the serum of hyperuricaemic rats (*P* < 0.05). In contrast, the green tea group and Ben groups’ BUN levels showed no considerable reduction. However, the BUN levels were significantly reduced in the yellow tea group.

### Effect of tea on the liver of rats with hyperuricaemia

**Fig. 2:**
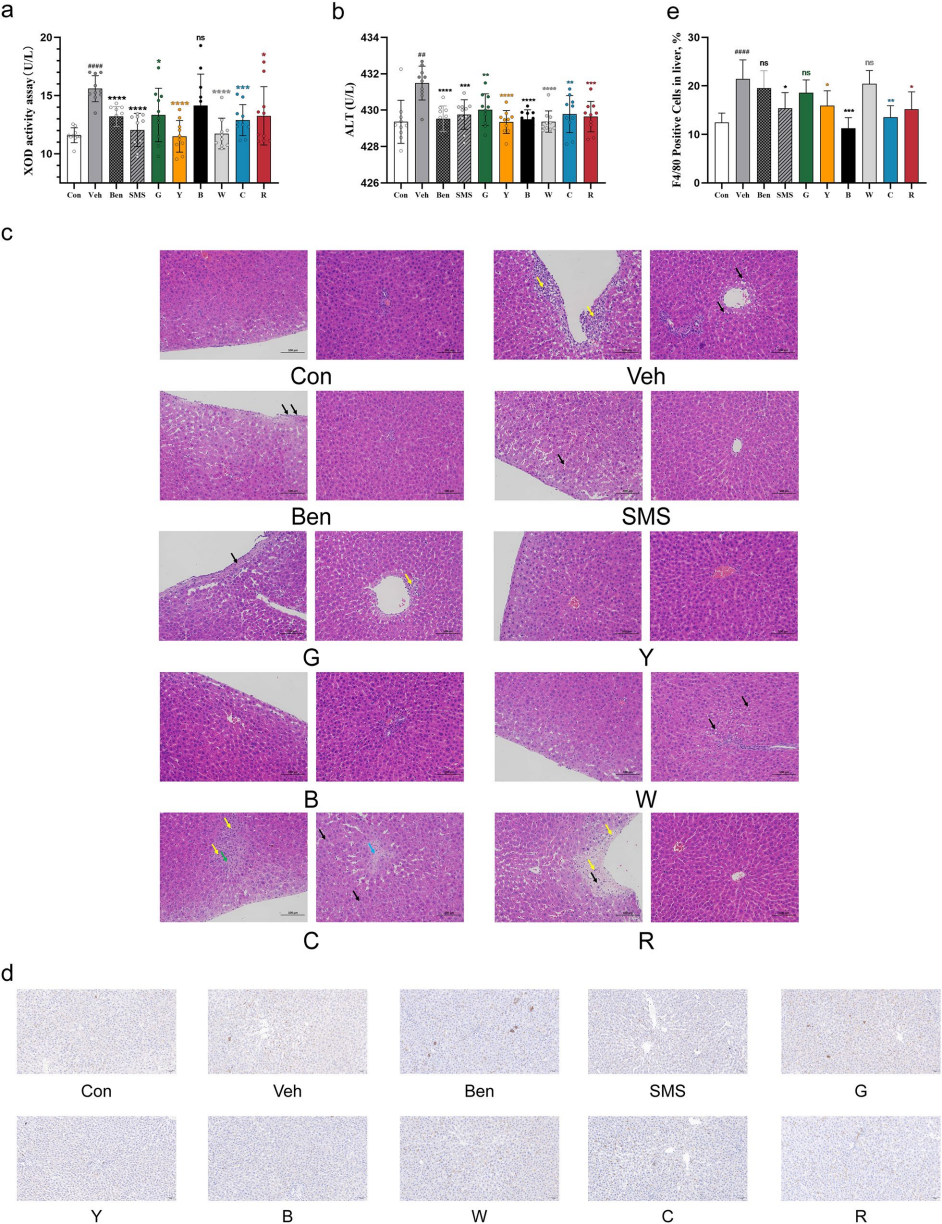
Effect of tea on the liver of rats with hyperuricaemia. **a** Effects of teas on hepatic XOD activity and **b** Serum ALT in PO- and hypoxanthine-induced hyperuricaemia in rats. Data are given as the mean ± SD (n = 10). **c** Hematoxylin and eosin (H&E)-stained liver tissues. Scale bars, 100 μm. The yellow, black, green, orange and blue arrows indicate inflammatory infiltration, hepatocyte steatosis, hepatocyte death, fibroproliferative lesions around the central veins and capsular fibroproliferative lesions, respectively. **d** Immunohistochemical staining for F4/80 in liver tissue. Scale bars, 50 μm. **e** Quantification of the proportion of F4/80 -positive cells in the liver. ^#^Represents a significant difference compared with the Con group, ^##^*P* < 0.01; ^####^*P* < 0.0001; *represents a significant difference compared with the Veh group, *P < 0.05, **P < 0.01, ***P < 0.001,****P < 0.0001

XOD is a critical enzyme that promotes the production of uric acid. As shown in Fig. 2a, the serum XOD levels in the Veh group were significantly higher than those in the Con group. Except for the black tea group, the XOD levels of all intervention groups decreased. It is worth noting that yellow tea and white tea significantly reduced XOD levels (*P* < 0.0001), and their effects were similar to those of the SMS group. The injection of PO- and hypoxanthine increased the activity of serum ALT (Fig. 2b). The consumption of all kinds of tea significantly suppressed this increase in ALT activity (*P* < 0.01). The liver histological changes of the Congroup and experimental mice are depicted in Fig. 2c. The Veh group exhibited several characteristic histologic alterations, including inflammatory cell infiltration (yellow arrow) under the capsule, and hepatocyte steatosis (black arrow) around the local veins, and parenchyma. Some liver cells died and disappeared (green arrow), fibroproliferative lesions around the central veins (blue arrow) and capsular fibroproliferative lesions (orange arrow). The inflammatory cell infiltration and hepatocellular disease were alleviated in the tea treatment and positive control groups. In particular, the yellow tea and black tea significantly alleviated the pathological lesions induced by PO- and hypoxanthine. To further confirm inflammatory infiltration, F4/80 (a marker of macrophages)was detected. The number of F4/80-positive cells was significantly increased in the livers of hyperuricaemic rats and this effect was abolished by yellow tea (*P* < 0.05), red tea(*P* < 0.05), cyan tea *(P* < 0.01),and especially black tea *(P* < 0.001)(Fig. 2d, e).Interestingly, benzbromarone did not inhibit the infiltration of macrophages in the liver of hyperuricaemic rats, while all teas except white tea and Simiao San did.

### Effect of tea on the kidneys of rats with hyperuricaemia

**Fig. 3:**
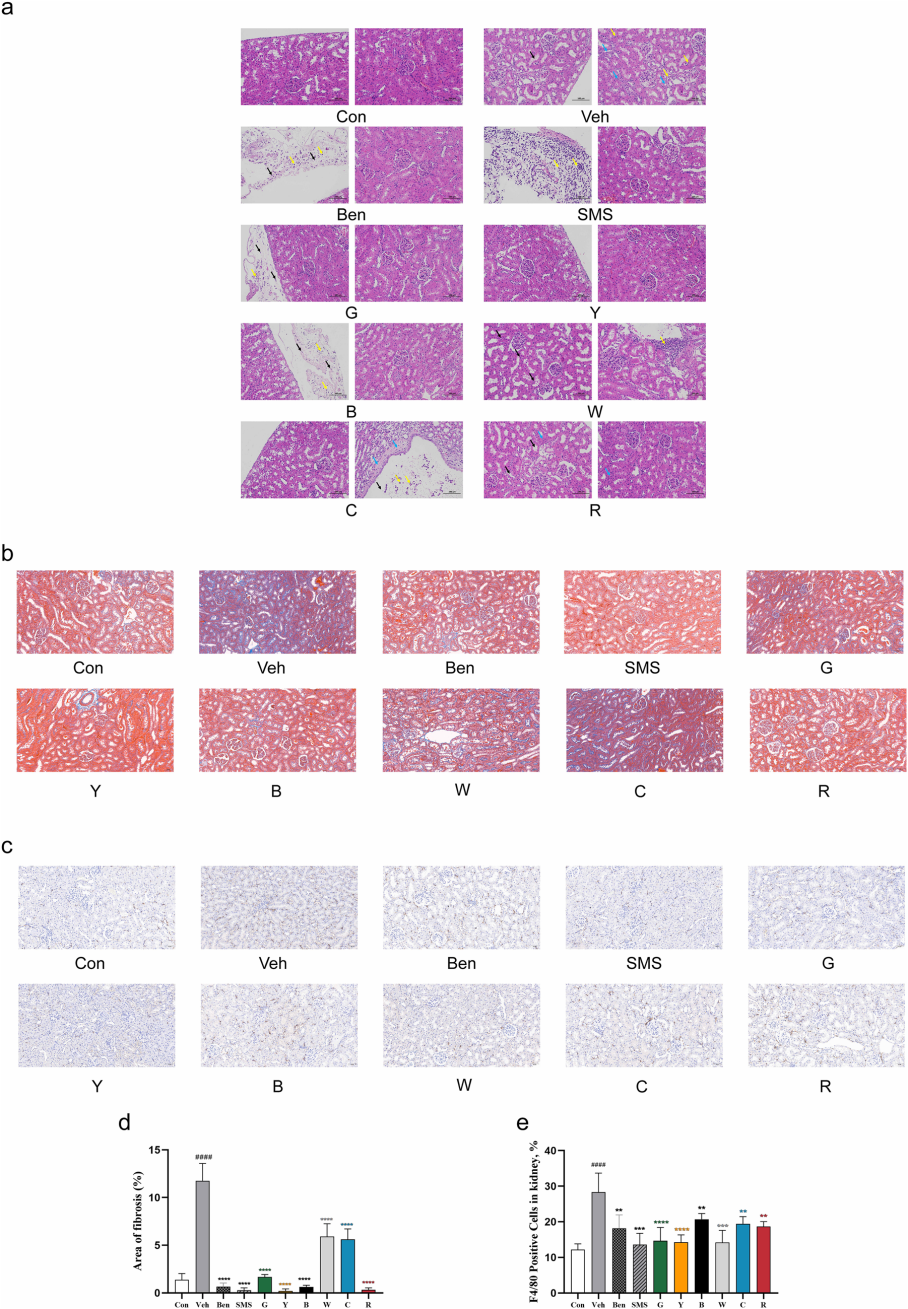
Effect of tea on histopathology of rat kidney induced by PO- and hypoxanthine. The right kidney of the rat was fixed, embedded in paraffin, sectioned, and stained. **a** H&E. Scale bars, 100 μm. The blue, black, and yellow arrows indicate conspicuous dilation of renal tubules, fibroproliferative lesions and inflammatory infiltration, respectively. **b** Masson’s trichrome. Scale bars, 50 μm. **c** Immunohistochemistryfor F4/80. Scale bars, 50 μm. **d** Bar diagrams represent the area of interstitial fibrosis (%). **e** Quantification of the proportion of F4/80 -positive cells in the kidney

The changes in the organizational structure of the kidney were observed as a pathological characteristic of hyperuricaemia. Renal histologic sections were stained with H&E and Masson trichrome. The renal tissues in control rats showed a normal morphology without any evidence of inflammation. The kidney in rats treated with PO- and hypoxanthine exhibited several characteristic histologic alterations, including conspicuous dilation of renal tubules (blue arrow), swelling and proximal tubule necrosis. Fibroproliferative lesions occurred in the local renal capsule (black arrow) and inflammatory cell infiltration (yellow arrow).Compared with the Veh group, the tubulointerstitial and glomerular lesions were alleviated in the tea treatment groups and the positive control group, and theproximal tubule cells and the cytoplasm were relatively normal. Yellow tea treatment in particular alleviated the PO- and hypoxanthine-induced pathological lesions (Fig. 3a). All six types of tea attenuated renal fibrosis in hyperuricaemic rats (*P* < 0.001), and yellow tea, red tea, and black tea completely blocked the formation of renal fibrosis(Fig. 3b, d).

To further confirm the inflammatory infiltration, F4/80 (a marker of macrophages) was detected. The number of F4/80-positive cells was significantly increased in the kidneys of hyperuricaemic rats, which was effectively abolished by black tea treatment *(P* < 0.01), cyan tea treatment *(P* < 0.01), red tea treatment *(P* < 0.01), white tea treatment *(P* < 0.001), and especially yellow tea and green tea treatment *(P* < 0.0001) (Fig. 3c, e).

### The candidate ingredients of yellow tea and its targets

The above results indicate that yellow tea can reduce the biochemical indicators and histopathological changes in hyperuricaemia rats. Therefore, we analysed and identified the ingredients in tea infusion by using LC–MS system. The representative base peak chromatograms (BPCs) of the six tea samples are shown in Additional file 1: Fig. S1. Based on the OB and DL parameters, 10 active compounds of yellow tea were identified (Table 1). The 9 flavonoids included two flavones, four flavonols, and three flavan-3-ols. By searching the HERB database and TCMID database, we obtained 730 potential targets of the 10 yellow tea compounds after removing the repetitive targets.

**Table 1:** The candidate ingredients of yellow tea and its targets

Compound ID	Compound name	Chemical structure	OB (%)	DL
5.301_458.08396	(-)-Epigallocatechin gallate		55.09	0.77
5.705_446.08371	Baicalein 7-O-Glucuronide		40.12	0.75
6.548_432.10466	Isovitexin		31.29	0.72
4.712_578.14173	Procyanidin B1		67.87	0.66
6.352_302.00551	Ellagic acid		43.06	0.43
8.341_302.04179	Quercetin		46.43	0.28
7.662_302.04178	Herbacetin		36.07	0.27
5.149_290.07823	Epicatechin		48.96	0.24
9.424_286.04692	Kaempferol		41.88	0.24
7.739_270.05195	Galangin		45.55	0.21

### Construction of the PPI network of the active ingredients of yellow tea and hyperuricaemia

**Fig. 4:**
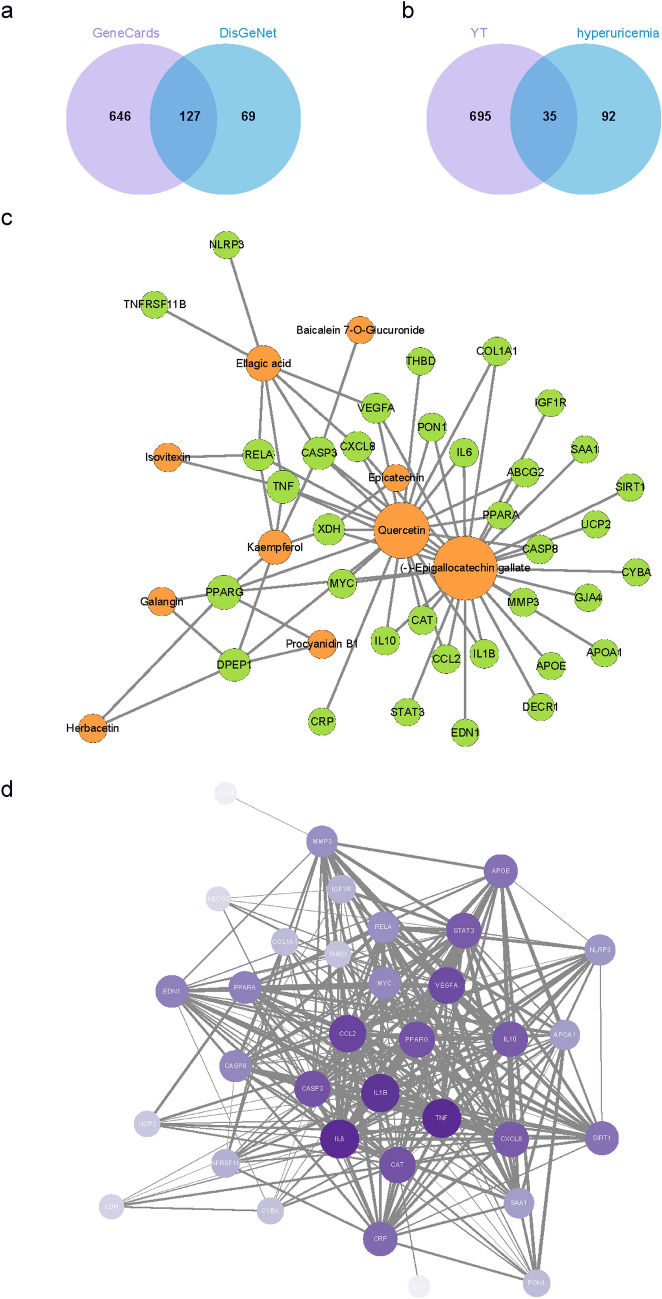
Screening of active ingredients related to hyperuricaemia in yellow tea. **a** Intersection targets related to hyperuricaemia from the GeneCards and DisGeNet databases. **b** The overlapping targets for yellow tea (YT) bioactive compounds and targets related to hyperuricaemia. **c** The active ingredient-target network for yellow tea. The orange node represents ingredient nodes, the green node represents predicted targets, and the connecting lines represent the interaction between the ingredients and the targets. The node size represents the corresponding degree value. **d** PPI network of the common targets of the active ingredients of yellow tea and hyperuricaemia

Hyperuricaemia-related targets were searched and retrieved from DisGeNET and GeneCards, including 196 and 773 targets, respectively. We finally retained 127 overlapping targets by taking the intersection (Fig. 4a). Thirty-five overlapping targets between yellow tea compounds and targets related to hyperuricaemia were obtained (Fig. 4b). Compounds-targets network of the 10 compounds from yellow tea and 35 targets was also constructed (Fig. 4c). These common targets were inputted into STRING to construct a PPI network, and the network was visualized using Cytoscape 3.9.0. The PPI network consisted of 41 nodes with 295 edges (Fig. 4d). The core targets of yellow tea for the treatment of hyperuricaemia were detected by MCODE and consisted of NLRP3 inflammasome (NLRP3), peroxisome proliferator-activated receptor-α (PPARA), IL-1β (IL1B), interleukin-6 (IL6), tumour necrosis factor (TNF), peroxisome proliferative activated receptor gamma (PPARG), C–C motif chemokine ligand 2 (CCL2), vascular endothelial growth factor A (VEGFA), and signal transducer and activator of transcription (STAT3) (Additional file 1: Table S1).

### GO analysis and KEGG pathway enrichment analysis of the common targets

**Fig. 5:**
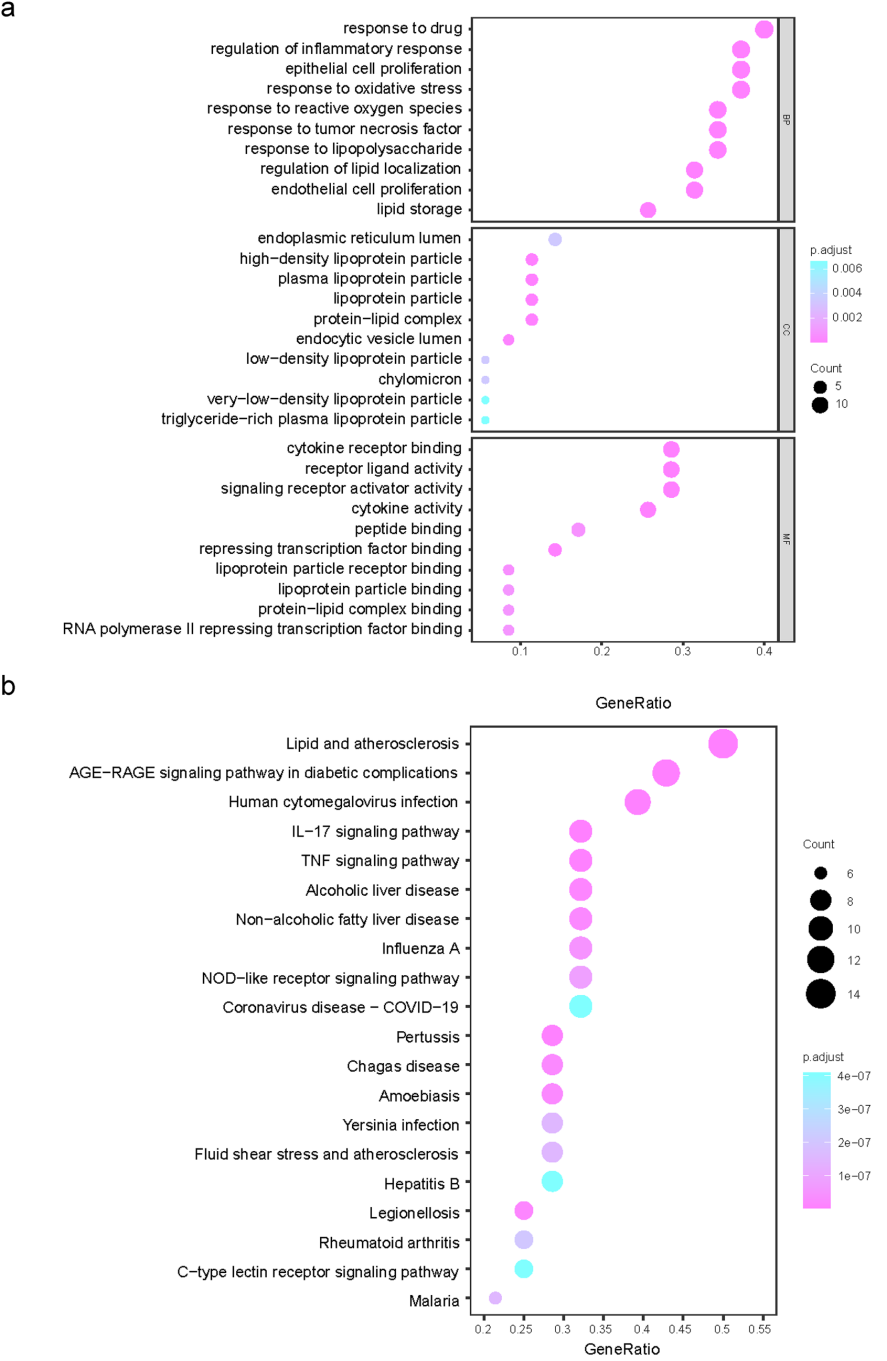
GO analysis and KEGG pathway enrichment analysis of common targets. **a** Bubble diagram of GO enrichment of common targets. **b** KEGG pathway analysis of common targets. Gene ratio refers to the percentage of enriched targets to all targets, and the counts refer to the number of enriched targets

To investigate the functions of these genes, we performed GO annotation and KEGG pathway enrichment analysis. GO enrichment analysis was used to identify the potential biological processes (BP), molecular functions (MF), and cellular components (CC) of the 35 genes of yellow tea against hyperuricaemia. According to the criteria of adjusted *p *value < 0.05 and *q *value < 0.05, we generated 1482 GO terms, including 1414 BP-, 15 CC-, and 52 MF-related GO terms. The top 10 GO terms for BP, CC, and MF are shown in Fig. 5a. Enrichment was observed in the BP: the regulation of inflammatory response, epithelial cell proliferation, response to oxidative stress, response to reactive oxygen species, response to tumour necrosis factor, response to lipopolysaccharide, regulation of lipid localization, endothelial cell proliferation, and lipid storage. Enrichment was observed in the MF: cytokine receptor binding, receptor–ligand activity, signalling receptor activator activity, cytokine activity, peptide binding, repressing transcription factor binding, lipoprotein particle binding, protein-lipid complex binding, and RNA polymerase II transcription factor activity. KEGG enrichment analysis was employed to identify the potential cellular signalling pathways. Ninety-four KEGG pathways were significantly enriched to reveal the possible molecular mechanism by which yellow tea prevents hyperuricaemia. The top 20 KEGG pathways are illustrated in Fig. 5b. Among those enriched, the signalling pathways associated with hyperuricaemia include TNF, IL-17, lipid and atherosclerosis, and the AGE-RAGE signalling pathway, suggesting that yellow tea may play a role in treating hyperuricaemia mainly through the regulation of the above signalling pathways.

### Teas alleviated the inflammatory response in hyperuricaemic rats

**Fig. 6:**
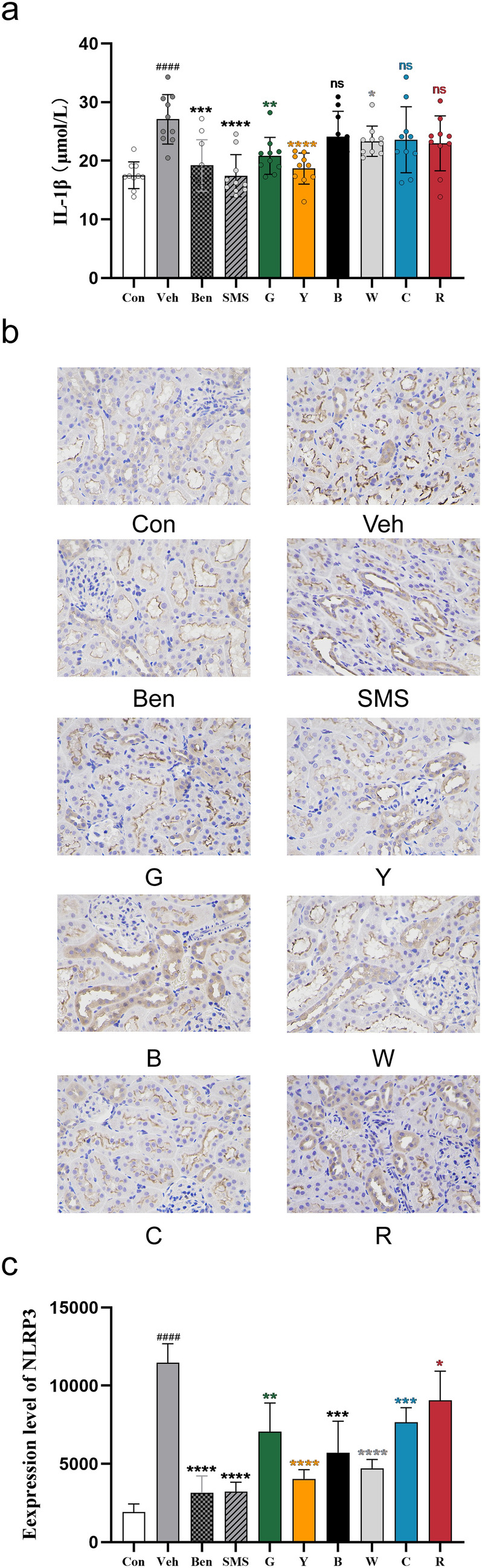
Teas alleviated the inflammatory response in hyperuricaemia rats. **a** Serum levels of IL-1β were measured with an ELISA kit. Data are shown as the means ± sem (n = 10 rats). **b** Immunohistochemical analysis of NLRP3 was performed in kidney tissue. Scale bars, 20 μm. **c** Quantification of the areal density of NLRP3 in the kidney

The above experimental results indicate that yellow tea may reduce hyperuricaemia in rats by inhibiting inflammation. Therefore, we detected the levels of proinflammatory factors IL-1β in serum and NLRP3 expression in the kidney. As shown in Fig. 6a, black tea, cyan tea, and red tea had no significant effect on the level of IL-1β in the serum of hyperuricaemic rats. White tea and green tea slightly reduced it, while yellow tea significantly reduced the level of IL-1β in the serum of rats with hyperuricaemia. We found that all six kinds of tea could downregulate the expression of NLRP3 protein in the kidneys of hyperuricaemic rats, especially yellow and white tea (Fig. 6b, c). These data suggest that yellow tea may alleviate hyperuricaemia in rats by inhibiting NLRP3 inflammasome activation.

## Discussion

Diet has a stimulating effect on hyperuricaemia and even gout. Our study investigated the effect of different types of tea on hyperuricaemia in rats. We evaluated the biochemical indicators of uric acid and explored the effects of different teas on the liver and kidney of rats with hyperuricaemia. Our study is the first to compare the impact of various teas on hyperuricaemia in rats. Here, we showed that drinking green tea cannot reduce the uric acid level in the serum of rats with hyperuricaemia, which is consistent with the results of a previous study by Zhang [34]. Other types of tea can reduce the serum uric acid level of hyperuricaemic rats to varying degrees. However, this finding is inconsistent with a previous cross-sectional study showing that frequent green tea consumption did not increase the risk of hyperuricaemia compared with a ‘no intake’ reference group [39]. More interestingly, we found that black and yellow tea could significantly reduce the levels of various biochemical indicators (UA, CRE, BUN, XOD) of hyperuricaemia. Yellow tea, in particular, can reduce biochemical indicators and reverse liver and kidney damage.

Yellow tea was the second tea manufactured after green tea was invented in China, and its history can be dated back to the mid-Tang Dynasty in 618–907 CE [40]. Unlike green tea, yellow tea undergoes a special process called “sealed yellowing”, the key process to create the unique characteristics of yellow tea, in which the green colour of the fresh leaves turns into yellow, and the characteristics of the tea also changes [41]. Yellow tea has been demonstrated to have a protective effect against cardiovascular diseases and liver injury [42, 43] via a number of different mechanisms. Currently, there are few reports on the protective effect of yellow tea against renal diseases. Our data show that the natural compounds in yellow tea are likely to be good candidates for dietary supplements or drugs to treat hyperuricaemia.

A variety of active ingredients in tea have been confirmed to have anticancer, antiobesity and reversing steatohepatitis effects. We identified and analysed the candidate components of yellow tea and obtained 35 targets related to hyperuricaemia based on LC–MS/MS. Our research also examined the possible targets and participating signalling pathways by GO analysis and KEGG pathway enrichment analysis. The active ingredients in yellow tea participate in many cell signalling pathways by binding to different proteins, such as TNF, atherosclerosis, and the AGE-RAGE signalling pathway. Our experiments showed that green tea and white tea could slightly reduce the proinflammatory cytokines IL-1βinhyperuricaemia rats; however, yellow tea could significantly reduce its level.

Epigallocatechin gallate (EGCG) attenuates microglial inflammation by inhibiting inflammasome activation [44]. Procyanidin B1 inhibited the apoptosis of oocytes and cumulus cells by reducing oxidative stress [45]. These ingredients may exert the anti-inflammatory and antioxidant effects of tea. We did not distinguish the specific actual impact of every compound because of the lack of standards for the quantitative analysis of these active compounds. Nevertheless, our results may provide more details for studying the mechanism of hyperuricaemia and the efficacy of tea in the prevention or possible treatment of hyperuricaemia and provide a reference for patients with hyperuricaemia to drink tea.

## Conclusions

To our knowledge, this is the first report that compared the effects of six types of tea on hyperuricaemia and found that green tea cannot reduce the serum uric acid level of hyperuricaemic rats. Compared with other teas, yellow tea can significantly improve hyperuricaemia, which may occur by regulating the inflammatory response, autophagy, and apoptosis. Therefore, this study provides a potential candidate for the treatment of hyperuricaemia and a basis for selecting therapeutic tea for patients with hyperuricaemia.

## Supplementary Information

**Additional file 1: Table S1.** The core targets of yellow tea for the treatment of hyperuricaemia. **Figure S1.** Representative base peak chromatogram (BPCs) of (a) green tea, (b) yellow tea, (c) black tea, (d) cyan tea, (e) white tea, and (f) red tea in negative ion mode.

## Data Availability

The datasets used and/or analysed during the current study are available from the corresponding author on reasonable request.

## Abbreviations

PO: Potassium oxonate
UA: Uric acid
ALT: Alanine aminotransferase
XOD: Xanthine oxidase
BUN: Blood urea nitrogen
CRE: Creatinine
ELISA: Enzyme-linked immunosorbent assay
IL-1β: Interleukin-1β
LC–MS/MS: Liquid chromatography tandem mass spectrometry
MSU: Monosodium urate
TCM: Traditional Chinese medicine
IHC: Immunohistochemistry
NLRP3: NOD-like receptor protein 3
ADMET: Absorption, distribution, metabolism, excretion, and toxicity
TCMSP: Traditional Chinese Medicine Systems Pharmacology Database and Analysis Platform
MCODE: Molecular complex detection
GO: Gene ontology
KEGG: Kyoto Encyclopedia of Genes and Genomes
PPI: Protein–protein interaction
STRING: Search tool for recurring instances of neighbouring genes
BP: Biological processes
MF: Molecular functions
CC: Cellular components
EGCG: Epigallocatechin gallate
