# Supplementary material for: Potential role of tea drinking in preventing hyperuricaemia in rats: biochemical and molecular evidence
Source: Chin Med. 2022 Sep 15;17:108. doi: 10.1186/s13020-022-00664-x (PMC9479443; doi:10.1186/s13020-022-00664-x)
Supplement: Supplementary file 1 — Additional file 1: Table S1. The core targets of yellow tea for the treatment of hyperuricaemia. Figure S1. Representative base peak chromatogram (BPCs) of (a) green tea, (b) yellow tea, (c) black tea, (d) cyan tea, (e) white tea, and (f) red tea in negative ion mode. [file 13020_2022_664_MOESM1_ESM.docx]

**Table S1.** The core targets of yellow tea for the treatment of hyperuricaemia.

| MCODE:: Clusters | MCODE:: Node Status | MCODE:: Score | Name | Selected | Shared name |
| --- | --- | --- | --- | --- | --- |
| Cluster 0 | Seed | 14 | NLRP3 | TRUE | NLRP3 |
| Cluster 0 | Clustered | 14 | PPARA | TRUE | PPARA |
| Cluster 0 | Clustered | 13.88333333 | EDN1 | TRUE | EDN1 |
| Cluster 0 | Clustered | 13.81578947 | CASP3 | TRUE | CASP3 |
| Cluster 0 | Clustered | 13.81578947 | CCL2 | TRUE | CCL2 |
| Cluster 0 | Clustered | 13.81578947 | CXCL8 | TRUE | CXCL8 |
| Cluster 0 | Clustered | 13.81578947 | IL10 | TRUE | IL10 |
| Cluster 0 | Clustered | 13.81578947 | IL1B | TRUE | IL1B |
| Cluster 0 | Clustered | 13.81578947 | IL6 | TRUE | IL6 |
| Cluster 0 | Clustered | 13.81578947 | SIRT1 | TRUE | SIRT1 |
| Cluster 0 | Clustered | 13.81578947 | STAT3 | TRUE | STAT3 |
| Cluster 0 | Clustered | 13.81578947 | TNF | TRUE | TNF |
| Cluster 0 | Clustered | 13.81578947 | VEGFA | TRUE | VEGFA |
| Cluster 0 | Clustered | 13.76666667 | CASP8 | TRUE | CASP8 |
| Cluster 0 | Clustered | 13.69117647 | APOE | TRUE | APOE |
| Cluster 0 | Clustered | 13.69117647 | CRP | TRUE | CRP |
| Cluster 0 | Clustered | 13.0994152 | CAT | TRUE | CAT |
| Cluster 0 | Clustered | 13.01754386 | PPARG | TRUE | PPARG |
| Cluster 0 | Clustered | 12.87619048 | RELA | TRUE | RELA |
| Cluster 0 | Clustered | 12.675 | MYC | TRUE | MYC |
| Cluster 0 | Clustered | 12 | SAA1 | TRUE | SAA1 |
| Cluster 0 | Clustered | 11.86813187 | MMP3 | TRUE | MMP3 |


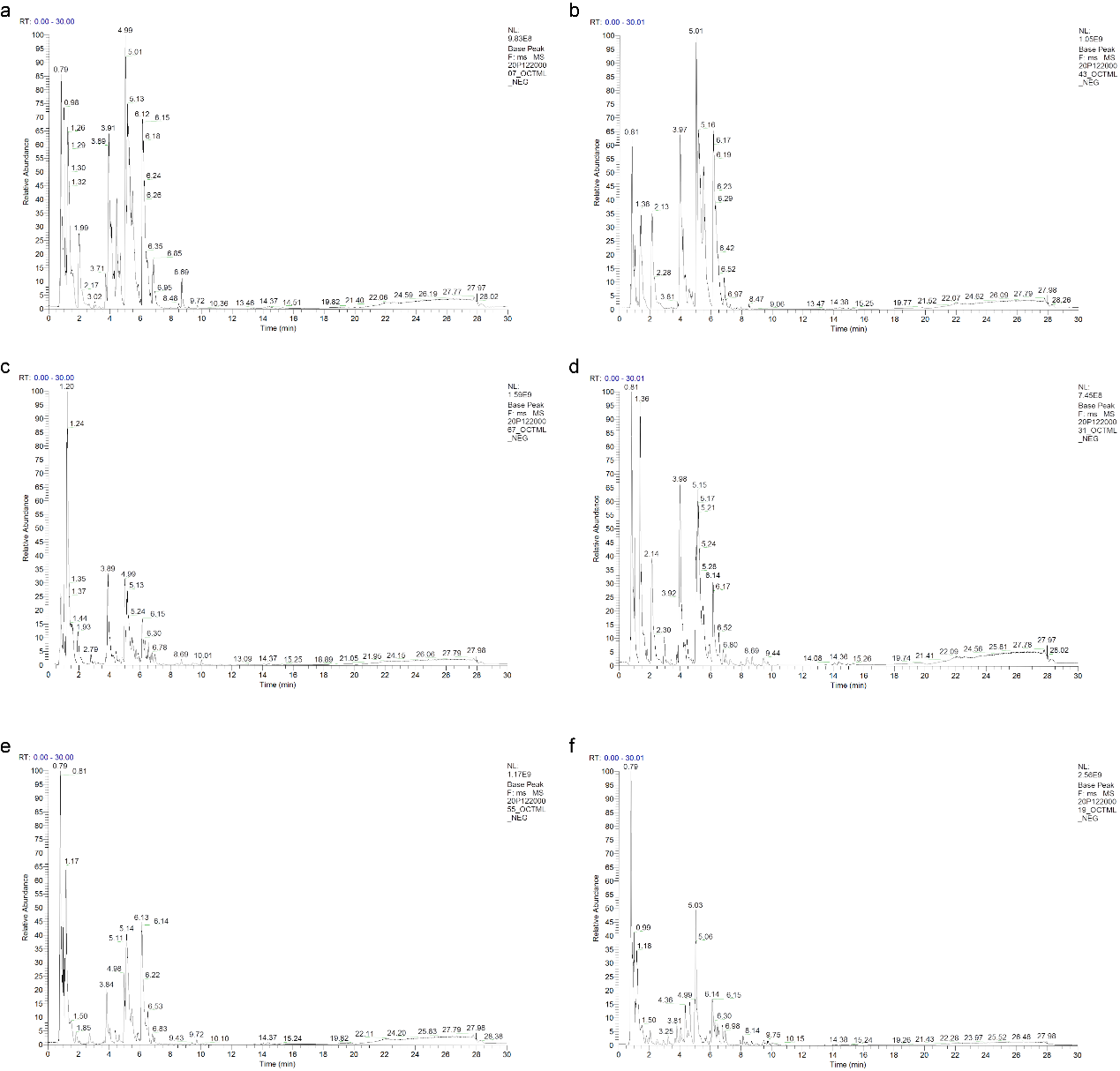


**Figure S1.** Representative base peak chromatogram (BPCs) of (a) green tea, (b) yellow tea, (c) black tea, (d) cyan tea, (e) white tea, and (f) red tea in negative ion mode.
